# Common microRNA regulated pathways in Alzheimer’s and Parkinson’s disease

**DOI:** 10.3389/fnins.2023.1228927

**Published:** 2023-09-01

**Authors:** Betina Awuson-David, Adrian C. Williams, Benjamin Wright, Lisa J. Hill, Valentina Di Pietro

**Affiliations:** ^1^School of Biomedical Sciences, Institute of Clinical Sciences, University of Birmingham, Birmingham, United Kingdom; ^2^Department of Neurology, University Hospitals Birmingham NHS Foundation Trust, Birmingham, United Kingdom; ^3^Institute of Inflammation and Ageing, University of Birmingham, Birmingham, United Kingdom

**Keywords:** microRNA, neurodegeneration, Alzheimer’s disease, Parkinson’s disease, biomarkers, therapeutic targets

## Abstract

MicroRNAs (miRNAs) are small non-coding RNAs involved in gene regulation. Recently, miRNA dysregulation has been found in neurodegenerative diseases such as Alzheimer’s disease (AD) and Parkinson’s disease (PD). The diagnosis of Alzheimer’s and Parkinson’s is currently challenging, mainly occurring when pathology is already present, and although treatments are available for both diseases, the role of treatment is primarily to prevent or delay the progress of the diseases instead of fully overcoming the diseases. Therefore, the challenge in the near future will be to determine effective drugs to tackle the dysregulated biological pathways in neurodegenerative diseases. In the present study, we describe the dysregulation of miRNAs in blood of Alzheimer’s and Parkinson’s patients with the aim to identify common mechanisms between the 2 pathologies and potentially to identify common therapeutic targets which can stop or delay the progression of two most frequent neuropathologies. Two independent systematic reviews, bioinformatic analysis, and experiment validation were performed to identify whether AD and PD share common pathways. A total of 15 common miRNAs were found in the literature and 13 common KEGG pathways. Among the common miRNAs, two were selected for validation in a small cohort of AD and PD patients. Let-7f-5p and miR-29b-3p showed to be good predictors in blood of PD patients.

## Introduction

Neurodegeneration is a term that can be applied to several conditions which share, as common features, the progressive loss of structure and function of central nervous system (Wareham et al., 2022). Neurodegenerative diseases include a variety of pathological disease entities and clinical presentations, among these, the most prevalent are Alzheimer’s disease (AD, progressive dementia), Parkinson disease (PD, progressive movement disorder with or without dementia; Poewe et al., 2017; Leng and Edison, 2021). Although AD and PD are two distinct diseases they often appear together and share several risk factors contributing to the development and progression of the two pathologies, including aging, genetic and epigenetic factors, environmental factors (Poirier et al., 1993; Hou et al., 2019; Businaro et al., 2021; Marques-Aleixo et al., 2021). AD and PD are neurodegenerative diseases which affect memory, movement and communication. PD typically presents clinically with a tremor at rest, Bradykinesia, rigidity and gait impairments with pathological features of dopaminergic neuron loss and presence of Lewy bodies, whereas AD typically presents clinically as learning and memory loss, reductions in executive function and speech and, pathologically, is associated with the presence of amyloid-beta protein and neurofibrillary tangles. Despite their clinical and pathological features, there are common pathological mechanisms which occur. These include disturbances in iron metabolism (Oshiro et al., 2011), build-up α-Synuclein and Tau protein (Kim et al., 2004; Sengupta and Kayed, 2022), higher levels of oxidative stress and mitochondrial dysfunction (Alqahtani et al., 2023), reductions of noradrenergic neurons in the Locus Coeruleus and increased levels of neuroinflammation (Sakakibara et al., 2021; Zhou et al., 2021). However, despite the considerable effort in studying these two pathologies, our understanding of the mechanisms involved and the link between them remains rudimentary at best.

With this study, we investigated the commonalities of these two pathologies looking at similarities and differences in microRNA (miRNA, miR) expression patterns and pathways regulated by them. The emerging developments in the field of miRNAs has led to the investigation of their function in the nervous system physiology and pathology. High throughput sequencing experiments have reported that almost 50% of the miRNAs are expressed in the mammalian brain (Shao et al., 2010) and can play a role locally in synaptic activity, neuronal connectivity and neuroplasticity (Aksoy-Aksel et al., 2014; Ryan et al., 2015).

There is also an increased interest in miRNA roles in neurodegenerative conditions. In AD it has been reported that Aβ production involves miRNA-mediated regulation and that dysregulation of miR-29a/29b-1 alters the expression of β-secretase (BACE-1) (Hébert et al., 2008; Wang W. X. et al., 2008; Geekiyanage and Chan, 2011). MiRNAs have also been found to play an important role in PD, for example, dysregulation of miR-7 and miR-34b/c was associated with PD mitochondrial dysfunction and oxidative stress (Junn et al., 2009; Miñones-Moyano et al., 2011). Additionally, miR-133b, which plays a key role in the differentiation of dopaminergic neurones, was found to be downregulated in PD (Kim et al., 2007).

This review has the aim to identify the common features of two most common neurodegenerative diseases, AD and PD, improving our understanding of how critical biological pathways impact AD and PD and influence the neurodegenerative processes and the clinical outcomes. Common and altered AD and PD pathways can also be used to identify promising new targets for drug development as well as to identify new molecules as non-invasive biomarkers.

## Results

### Search results

**Figure 1:**
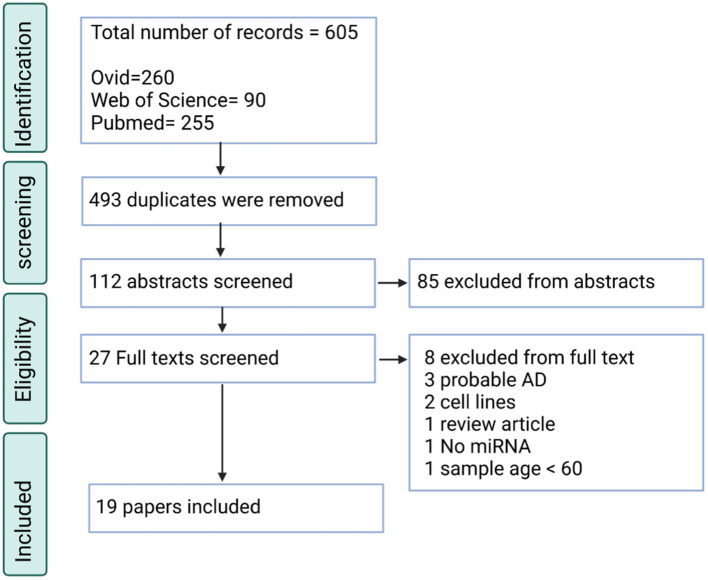
PRISMA flow chart for the AD systematic review displaying the screening process utilized to acquire the articles containing miRNAs.

There were two separate searches conducted for AD and PD in the systematic review. Through PubMed, EMBASE and Web of Science, 605 searches were retrieved for the AD search. 112 of these entries were unique, without duplicates. Through screening against the inclusion and exclusion criteria, 19 papers were included (Figure 1). Of the papers excluded, many did not describe AD and looked at other neurodegenerative disorders or mild cognitive impairment (*n* = 31). Also, many papers used animal models, which were not relevant to this study (*n* = 22). Lastly, another large reason for exclusion was that many of the papers were review articles, and this study required primary articles for analysis (*n* = 11).

**Figure 2:**
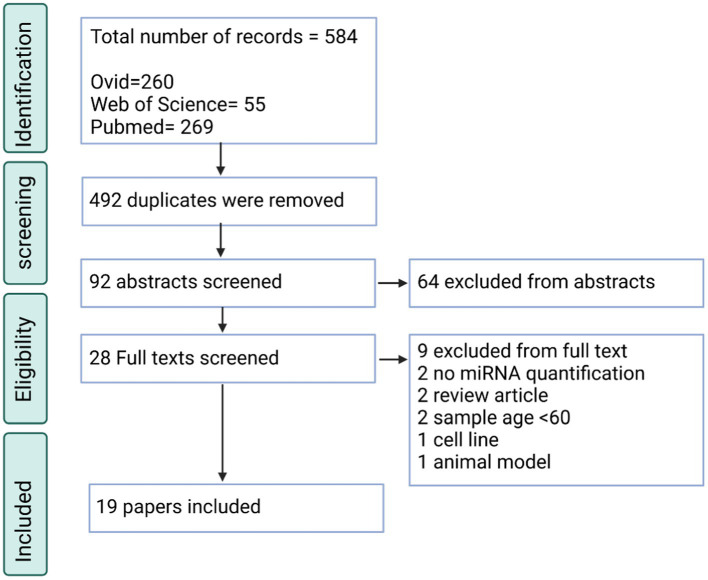
PRISMA flow chart for the PD systematic review displaying the screening process utilized to acquire the articles containing miRNAs.

Regarding the PD searches, 584 total entries were inputted, of which 92 were unique. Through screening, the total included papers were 19 papers altogether (Figure 2). The significant reasons for exclusion were similar to the AD searches. For this search, it was also found that many papers did not describe PD (*n* = 28), some were review articles (*n* = 17), and some did not quantify the miRNAs in the blood sample (*n* = 17).

### Data extraction

From the two independent systematic reviews, data regarding the sample population and findings were extracted (Tables 1 2). 19 papers describing blood biomarkers in AD and 19 in PD were identified through two independent systematic reviews. The extraction of these data allows the research question to be investigated, further analyzing if there are any common miRNAs between the two diseases. 15 common dysregulated miRNAs, including 5 showing a different directionality in the 2 pathologies, were finally found (Table 3). A problem encountered at this stage is that not all papers described the study population in fullness, some did not mention how many males and females there were and did not identify the severity of the AD or PD. Where the severity of the disease was not specified, it was assumed they had late or severe, and any other information which was unavailable was written as “NA.”

**Table 1:** Characteristics of the AD studies.

Author	AD group N Age Gender	Control group N Age Gender	AD severity	Upregulated miRNA	Downregulated miRNA	Sample type	Method
De Felice et al. (2020)	18 70.8 12F 6M	30 70.8 12F 6M	Moderate	miR-567	NA	Serum	RT-qPCR
Cheng et al. (2015)	39 78.6 23F 14M	59 75.8 31F 27M	Moderate	miR-361-5p miR-30e-5p miR-93-5p miR-15a-5p miR-143-3p miR-335-5p miR-106b-5p miR-101-3p miR-424-5p miR-106a-5p miR-18b-5p miR-3065-5p miR-20a-5p miR-582-5p	miR-1306-5p miR-342-3p miR-15b-3p	Serum	Deep sequencing & RT-qPCR
Wang et al. (2018)	20 71.3 13F 7M	20 70.8 10F 10M	Moderate	miR-1908	NA	Plasma	RT-qPCR
Han et al. (2020)	33 69.87 NA	30 65.35 NA	Severe	NA	miR-22	Serum	RT-qPCR
Kumar et al. (2017)	10 75.3 5F 5M	14 63.8 8F 6M	Mild & Moderate	miR-455-3p miR-3613-3p miR-4668-5p miR-5001-5p miR-4674 miR-4741	miR-122-5p	Serum	microarray
Kim et al. (2021)	56 73 28F 28M	67 73 33F 34M	Moderate	miR-1273 g-3p	NA	Plasma	RT-qPCR
Hojati et al. (2021)	18 NA NA	18 NA NA	Severe	miR-494-3p	miR-661	Serum	RT-qPCR
Dong Z. et al. (2021)	28 77.8 24F 4M	28 74.8 24F 4M	Severe	miR-22-3p miR-378a-3p miR-22-5p	miR-30b-5p miR-375-3p	Serum	NSG RT-qPCR
Siedlecki-Wullich et al. (2019)	56 77.77 41F 15M	14 68.29 7F 7M	Moderate	miR-92a-3p miR-181c-5p miR-210-3p	NA	Plasma	RT-qPCR
Hara et al. (2017)	27 74.7 NA	18 73.7 NA	Moderate	let-7f-5p miR-26b-5p	miR-501-3p	Serum	RT-qPCR
Serpente et al. (2020)	40 79 25F 15M	40 66.6 18F 22M	Severe	miR-23a-3p miR-223-3p miR-190a-5p	miR-100-3p	Plasma from neuronal derived extracellular vesicles	RT-qPCR
Heydari et al. (2021)	21 50–95 10F 11M	23 50–95 15F 8M	Severe	miR-324-3p	miR-331-3p	Serum	RT-qPCR
Mancuso et al. (2019)	40 79 22F 18M	40 75 24F 16M	Mild & Moderate	NA	miR-223-3p	Serum	RT-qPCR
Ludwig et al. (2019)	145 72.85 69F 76M	214 68.25 111F 103M	Mild & Moderate	miR-103a-3p miR-107 miR-532-5p miR-17-3p miR-1468-5p	miR-532-5p miR-17-3p	Blood sample	RT-qPCR
Lugli et al. (2015)	35 64.94 17F 18M	35 64.77 20F 15M	Severe	miR-548at-5p miR-138-5p miR-5001-3p miR-361-3p	miR-185-5p miR-342-3p miR-141-3p miR-548at-5p miR-342-5p miR-4772-3p miR-23b-3p miR-29b-3p miR-3916 miR-125b-5p miR-338-3p miR-3065-5p miR-139-5p miR-152-3p miR-150-5p miR-3613-3p	Plasma enriched in exosomes	RT-qPCR
Madadi et al. (2022)	56 73.9 35F 21M	50 71.36 26F 24M	Moderate	NA	miR-106b	Serum	RT-qPCR
Dong L. H. et al. (2021)	86 70.6 NA	121 69 NA	Mild, Moderate & Severe	NA	mir-202	Serum	RT-qPCR
Zeng et al. (2017)	60 63.9 37F 23M	30 62.1 17F 13M	Mild & Moderate	NA	miR-222	Plasma	Microarray RT-qPCR
Jia et al. (2021)	519 69 259F 260M	534 68 278F 256M	Mild	miR-10a-5pmiR-26b-5p miR-451a-5p	miR-139-3p miR-143-3p miR-146a-5p miR-485-5p	Serum	NSG RT-qPCR

Data extraction contains: reference; patient information for participants in each study including number of participants (N), mean age, gender (M:F) for AD and control group; and up-and downregulation of miRNAs; sample type and analysis methods.

**Table 2:** Characteristics of the PD studies.

Author	PD group N Age Gender	Control group N Age Gender	PD severity	Upregulated miRNA	Downregulated miRNA	Sample type	Method
Bai et al. (2017)	80 64 12F 68M	80 63.3 16F 64M	Mild & Moderate	NA	miR-29a miR-29b miR-29c	Serum	RT-qPCR
Oliveira et al. (2020)	20 71.6 10F 10M	20 69.5 10F 10M	Moderate	NA	miR-146a miR-335-3p miR-335-5p	Serum	RT-qPCR
Grossi et al. (2021)	15 75.7 15F	14 78.5 14F	Mild	miR-34a-5p	NA	Plasma and extracellular vesicles	RT-qPCR
Chen et al. (2018)	25 64.96 9F 16M	25 64 9F 16M	Newly diagnosed	mir-27a	let-7a let-7f miR-142-3p miR-222	Plasma	RT-qPCR
Uwatoko et al. (2019)	28 68.97 15F 13M	28 63.18 13F 15M	Moderate	miR-19b-3p	miR-671-5p	Plasma	Microarray RT-qPCR
Zago et al. (2022)	61 66 24F 37M	58 65 23F 35M	Newly diagnosed	miR-150-5p miR-215-5p	miR-144-3p	Serum	RT-qPCR
Cao et al. (2017)	109 69.8 36F 73M	40 67.9 15F 25M	Includes all stages of PD	miR-19b	miR-195 miR-24	Serum	RT-qPCR
Han et al. (2020)	98 61.46 53F 45M	40 63.75 17F 23M	Mild	NA	miR-29a, mir-29b mir-29c	Serum	RT-qPCR
Ding et al. (2016)	106 60.1 45F 61M	91 60.7 46F 45M	Includes all stages	miR-195	miR-185 miR-15b miR-221 miR-181a	Serum	RT-qPCR
Cai et al. (2021)	22 NA NA	9 NA NA	Severe	miR-195-3p miR-195-5p	miR-23b-3p miR-30b-5p	Plasma and circulating exosomes	sequencing
Behbahanipour et al. (2019)	36 61.3 25F 11M	16 62.5 11F 5M	Includes all stages	miR-885-5p miR-17-5p	miR-361-5p	Peripheral blood mononuclear cells	RT-qPCR
Fazeli et al. (2020)	30 62.11 9F 21M	14 63.93 3F 11M	Mild & Moderate	miR-27a-3p	miR-27b-3p	Peripheral blood mononuclear cells	RT-qPCR
Ruf et al. (2021)	82 69.74 33F 49M	83 67.4 37F 46M	Severe	NA	mir-1915-3p-mir-3665 mir-4745	Serum	RT-qPCR sequencing
Vallelunga et al. (2021)	51 61 22F 28M	56 63 31F 25M	Severe	miR-339-5p	miR-96-5p	Serum	RT-qPCR
Li H. et al. (2020)	80 64.6 38F 42M	60 64 29F 31M	Mild	NA	miR-150	Serum	RT-qPCR
Mancuso et al. (2019)	28 74 10F 18M	40 75 24F 16M	Mild	miR-223-3p	NA	Serum	RT-qPCR
Li et al. (2021)	69 66.5 35F 34M	21 64 11F 10M	Newly diagnosed & advanced	miR-31 miR-214	NA	Serum	RT-qPCR
Barbagallo et al. (2020)	30 69.6 6F 24M	30 67.9 20F 10M	Mild& Moderate	let-7d miR-22 miR-23a miR-24 miR-142-3p miR-181c miR-191-miR-222	NA	Serum	RT-qPCR
Chen et al. (2017)	169 61.9 88F 81M	170 61.6 28F 142M	Mild	miR-4639-5p	NA	Plasma	RT-qPCR

Data extraction contains: reference; patient information for participants in each study including number of participants (N), mean age, gender (M:F) for AD and control group; and up-and downregulation of miRNAs; sample type and analysis methods.

**Table 3:** Common miRNAs between AD and PD from the two independent systematic reviews.

MiRNA	Dysregulation in AD	Dysregulation in PD
miR-361-3p	**Upregulated**	**Downregulated**
miR-335-5p	**Upregulated**	**Downregulated**
miR-15b-3p	Downregulated	Downregulated
miR-22-3p	**Downregulated**	**Upregulated**
miR-30b-5p	Downregulated	Downregulated
miR-181c-5p	Upregulated	Upregulated
let-7f-5p	**Upregulated**	**Downregulated**
miR-23a-3p	Upregulated	Upregulated
miR-223-3p	Upregulated	Upregulated
miR-23b-3p	Downregulated	Downregulated
miR-29b-3p	Downregulated	Downregulated
miR-150-5p	**Downregulated**	**Upregulated**
-miR-222-3p	Downregulated	Downregulated
miR-146a-5p	Downregulated	Downregulated
miR-185-5p	Downregulated	Downregulated

Whether the miRNA levels were found to be up/downregulated levels was identified from the previous studies. Bold means a different miRNA trend in the 2 pathologies.

### Pathway analysis

**Figure 3:**
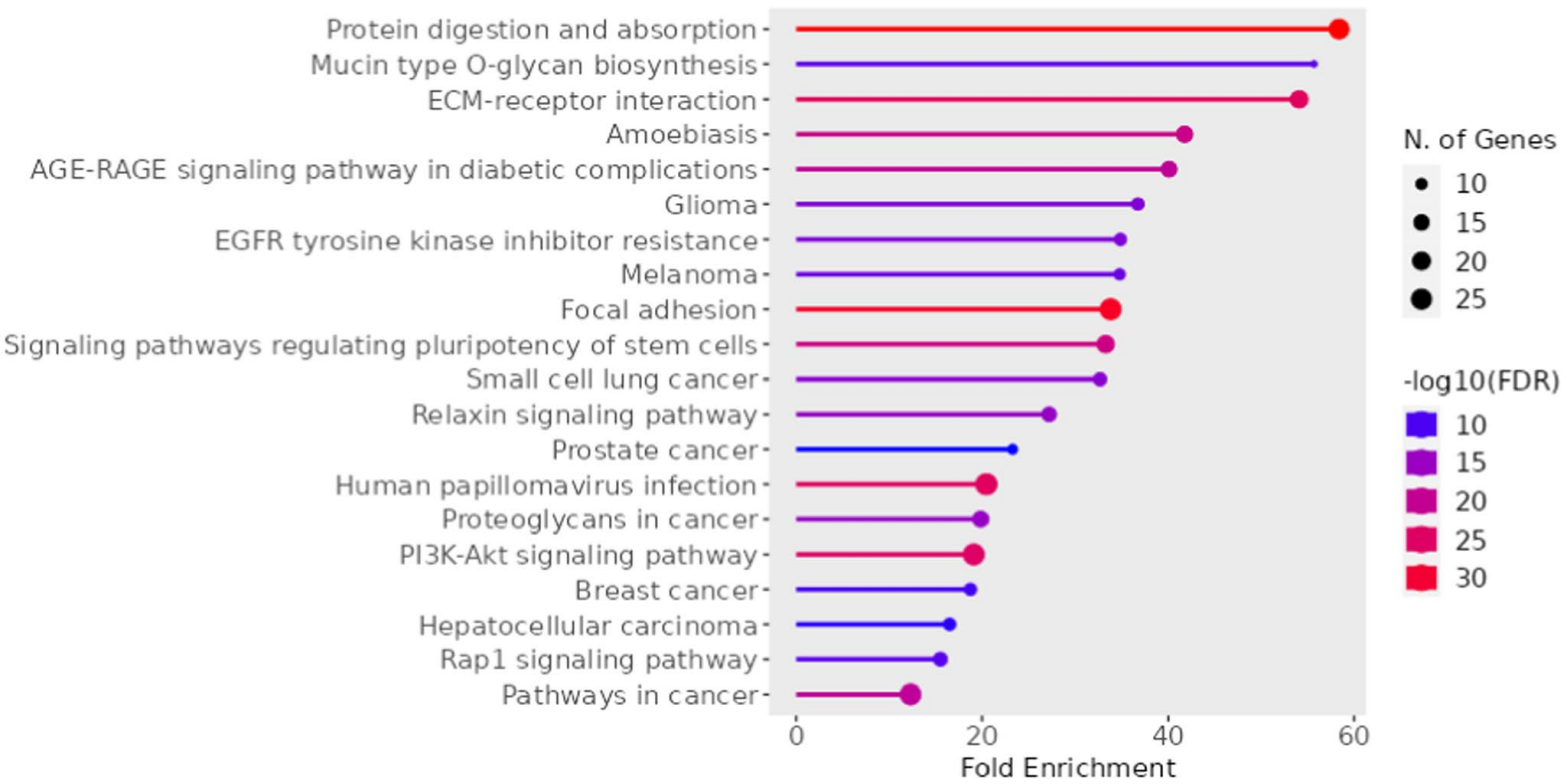
Chart diagram of gene enrichment analysis generated by ShinyGO.

The miRNAs identified for AD and PD were separately inputted into the DIANA database, observing pathways regulated by the miRNAs. The miRNAs obtained from the search regarding AD resulted in 162 KEGG pathways regulated by the miRNAs (Supplementary Table S1). To ensure the pathways are associated, a Fisher exact test was applied at *p* < 0.05. The most significantly affected pathways include the TGF β signaling pathway, Pancreatic secretion and the Calcium signaling pathway. The same was done for the systematic review regarding PD, which obtained 146 KEGG pathways (Supplementary Table S2). Some affected pathways include the ECM receptor interaction, Ras signaling pathways and PI3K-Akt pathway. The common miRNAs between PD and AD were also assessed to identify common pathways and gene targets, whereby 13 KEGG pathways were identified (Table 4). List of generated predicted targets was further evaluated using ShinyGO 0.77 software for enriched pathway and Gene Ontology (GO) analyses. A chart diagram generated with the list of predicted targets is represented in Figure 3. In addition, GO analyses for biological process, cellular component and molecular function are represented in Supplementary Tables S3–S5 respectively.

**Table 4:** The identified KEGG pathways associated with the common miRNAs, gene targets, and correspondent FDR-adjusted *p* values are also shown in this table.

KEGG pathway	miRNA	Gene targets	FDR-adjusted *p*-value
Fatty acid biosynthesis	miR-15b-5p miR-150-5p	ACSL4 ENSG00000068366 EHHADH ENSG00000113790	<1e-325
ECM-receptor interaction	miR-22-3p let-7f-5p miR-29b-3p miR-150-5p	SV2B ENSG00000185518 COL4A5 ENSG00000188153 COL24A1 ENSG00000171502 COL27A1 ENSG00000196739 ITGB6 ENSG00000115221 COL3A1 ENSG00000168542 SV2A ENSG00000159164 COL2A1 ENSG00000139219 COL4A2 ENSG00000134871 COL5A1 ENSG00000130635 COL1A1 ENSG00000108821 COL4A3 ENSG00000169031 COL4A4 ENSG00000081052 ITGA10 ENSG00000143127 COL1A2 ENSG00000164692 LAMC1 ENSG00000135862 ITGA7 ENSG00000135424 COL11A1 ENSG00000060718 COL6A3 ENSG00000163359 COL4A6 ENSG00000197565 GP9 ENSG00000169704 LAMA2 ENSG00000196569 COL5A3 ENSG00000080573 COL5A2 ENSG00000204262 COL4A1 ENSG00000187498 ITGB3 ENSG00000259207	<1e-325
Glycosphingolipid biosynthesis – lacto and neolacto series	miR-22-3p miR-23a-3p miR-23b-3p	FUT4 ENSG00000196371 B3GNT1 ENSG00000174684 B4GALT4 ENSG00000121578 FUT9 ENSG00000172461 ST8SIA1 ENSG00000111728	1.18299E-10
Fatty acid metabolism	miR-361-3p miR-15b-5p mir-15-3p	PTPLA ENSG00000165996 ACSL4 ENSG00000068366 EHHADH ENSG00000113790	1.24747E-10
Amoebiasis	let-7f-5p miR-29b-3p	ARG2 ENSG00000081181 COL4A5 ENSG00000188153 COL27A1 ENSG00000196739 SERPINB9 ENSG00000170542 COL3A1 ENSG00000168542 COL2A1 ENSG00000139219 CASP3 ENSG00000164305 COL4A2 ENSG00000134871 COL5A1 ENSG00000130635 COL1A1 ENSG00000108821 COL4A3 ENSG00000169031 PIK3R1 ENSG00000145675 COL4A4 ENSG00000081052 COL1A2 ENSG00000164692 LAMC1 ENSG00000135862 COL11A1 ENSG00000060718 COL4A6 ENSG00000197565	6.23466E-09
		LAMA2 ENSG00000196569 COL5A3 ENSG00000080573 IL10 ENSG00000136634 COL5A2 ENSG00000204262 PLCB4 ENSG00000101333 COL4A1 ENSG00000187498 PIK3R2 ENSG00000268173	
Mucin type O-Glycan biosynthesis	miR-30b-5p let-7f-5p miR-223-3p	GALNT7 ENSG00000109586 GCNT4 ENSG00000176928 GALNT18 ENSG00000110328 GALNT1 ENSG00000141429 GALNT3 ENSG00000115339 GALNT2 ENSG00000143641 C1GALT1 ENSG00000106392 GALNT16 ENSG00000100626	0.000010702
Glioma	miR-15b-5p mir-22-3p miR-29b-3p	BRAF ENSG00000157764 NRAS ENSG00000213281 CALM3 ENSG00000160014 IGF1R ENSG00000140443 CDK6 ENSG00000105810 AKT2 ENSG00000105221 PDGFB ENSG00000100311 PIK3R1 ENSG00000145675 PLCG2 ENSG00000197943 IGF1 ENSG00000017427 AKT3 ENSG00000117020 PTEN ENSG00000171862 PDGFA ENSG00000197461 PIK3R2 ENSG00000268173	0.000048596
Biosynthesis of unsaturated fatty acids	miR-361-3p	PTPLA ENSG00000165996	0.000917333
Fatty acid degradation	miR-15b-3p	ACSL4 ENSG00000068366 EHHADH ENSG00000113790	0.001529408
Signaling pathways regulating pluripotency of stem cells	miR-15b-5p let-7f-5p	DVL3 ENSG00000161202 NRAS ENSG00000213281 HOXB1 ENSG00000120094 ACVR1B ENSG00000135503 HAND1 ENSG00000113196 SMARCAD1 ENSG00000163104 IGF1R ENSG00000140443 FZD3 ENSG00000104290 FZD4 ENSG00000174804 RIF1 ENSG00000080345 SKIL ENSG00000136603 ACVR2A ENSG00000121989 ACVR1C ENSG00000123612 IGF1 ENSG00000017427 DUSP9 ENSG00000130829 PCGF3 ENSG00000185619 WNT9A ENSG00000143816	0.003325397
Protein digestion and absorption	miR-29b-3p	COL4A5 ENSG00000188153 ELN ENSG00000049540 COL27A1 ENSG00000196739 COL7A1 ENSG00000114270 COL22A1 ENSG00000169436	0.009310111
		COL3A1 ENSG00000168542 COL9A1 ENSG00000112280 COL21A1 ENSG00000124749 COL2A1 ENSG0000013921 COL15A1 ENSG00000204291 COL4A2 ENSG00000134871 COL5A1 ENSG00000130635 COL1A1 ENSG00000108821 COL4A3 ENSG00000169031 COL4A4 ENSG00000081052 SLC36A1 ENSG00000123643 COL1A2 ENSG00000164692 COL11A1 ENSG00000060718 COL6A3 ENSG00000163359 COL4A6 ENSG00000197565 COL5A3 ENSG00000080573 COL5A2 ENSG00000204262 -COL4A1 ENSG00000187498	
Lysine degradation	miR-29b-3p	SETDB2 ENSG00000136169 NSD1 ENSG00000165671 SETDB1 ENSG00000143379 DOT1L ENSG00000104885 WHSC1 ENSG00000109685 SUV420H2 ENSG00000133247	0.02030956
Focal adhesion	miR-15b-5p-miR-29b-3p	COL4A5 ENSG00000188153 COL27A1 ENSG00000196739. CAV2 ENSG00000105971 COL3A1 ENSG00000168542 AKT2 ENSG00000105221 COL2A1 ENSG00000139219 COL4A2 ENSG00000134871 COL5A1 ENSG00000130635 PDGFB ENSG00000100311 COL1A1 ENSG00000108821 COL4A3 ENSG00000169031 PIK3R1 ENSG00000145675 COL4A4 ENSG00000081052 COL1A2 ENSG00000164692 LAMC1 ENSG00000135862 IGF1 ENSG00000017427 PDGFC ENSG00000145431 COL11A1 ENSG00000060718 COL6A3 ENSG00000163359COL4A6 ENSG00000197565 LAMA2 ENSG00000196569 COL5A3 ENSG00000080573 VEGFA ENSG00000112715 PTEN ENSG00000171862 COL5A2 ENSG00000204262 COL4A1 ENSG00000187498 PDGFA ENSG00000197461 PIK3R2 ENSG00000268173	0.02849392

### Quality appraisal

For both systematic reviews, quality appraisals were performed on the papers that were used for the analysis (Table 5). As a result, all of the papers that were included were of a suitable quality. All scored a greater quality than 60%. All papers included had statistical analysis, a reliable methodology and overall low risk of bias. Some papers lacked a clear identification of confounding factors and also did not provide clear inclusion criteria for their study population.

**Table 5:** Quality appraisal using the JBI checklist.

	Q1 Clearly defined inclusion criteria	Q2 Detailed description of study population	Q4 Identification of confounding factors	Q5 strategies employed to minimize confounding factors	Q6 outcome measurement conducted reliably	Q7 appropriate statistical analysis conducted	Q8 low risk of bias
De Felice et al. (2020)	Y	Y	Y	Y	Y	Y	Y
Cheng et al. (2015)	Y	Y	Y	Y	Y	Y	Y
Wang et al. (2018)	N	Y	Y	Y	Y	Y	Y
Han et al. (2020)	Y	U	Y	Y	Y	Y	Y
Kumar et al. (2017)	Y	Y	N	Y	Y	Y	Y
Kim et al. (2021)	Y	Y	Y	Y	Y	Y	Y
Hojati et al. (2021)	Y	U	U	Y	Y	Y	Y
Dong Z. et al. (2021)	Y	Y	U	Y	Y	Y	Y
Siedlecki-Wullich et al. (2019)	Y	Y	Y	Y	Y	Y	Y
Hara et al. (2017)	Y	U	Y	U	Y	Y	Y
Serpente et al. (2020)	Y	Y	Y	Y	Y	Y	Y
Heydari et al. (2021)	U	Y	N	U	Y	Y	Y
Mancuso et al. (2019)	Y	Y	U	U	Y	Y	Y
Ludwig et al. (2019)	U	Y	Y	Y	Y	Y	Y
Lugli et al. (2015)	Y	Y	U	Y	Y	Y	Y
Madadi et al. (2022)	Y	Y	Y	Y	Y	Y	Y
Dong L. H. et al. (2021)	Y	N	Y	U	Y	Y	Y
Zeng et al. (2017)	Y	Y	U	Y	Y	Y	Y
Jia et al. (2021)	Y	Y	Y	Y	Y	Y	Y
Bai et al. (2017)	Y	Y	N	N	Y	Y	Y
Oliveira et al. (2020)	Y	Y	Y	Y	Y	Y	Y
Grossi et al. (2021)	Y	Y	Y	Y	Y	Y	Y
Chen et al. (2018)	Y	Y	Y	Y	Y	Y	Y
Uwatoko et al. (2019)	Y	Y	Y	Y	Y	Y	Y
Zago et al. (2022)	U	Y	Y	Y	Y	Y	Y
Cao et al. (2017)	Y	Y	Y	Y	Y	Y	Y
Han et al. (2020)	Y	Y	Y	Y	Y	Y	Y
Ding et al. (2016)	Y	Y	Y	Y	Y	Y	Y
Cai et al. (2021)	U	U	Y	Y	Y	Y	Y
Behbahanipour et al. (2019)	Y	Y	Y	Y	Y	Y	Y
Fazeli et al. (2020)	Y	Y	N	N	Y	Y	Y
Ruf et al. (2021)	N	Y	Y	Y	Y	Y	Y
Vallelunga et al. (2021)	Y	Y	Y	Y	Y	Y	Y
Li H. et al. (2020)	U	Y	Y	Y	Y	Y	Y
Mancuso et al. (2019)	Y	Y	Y	Y	Y	Y	Y
Li et al. (2021)	Y	Y	Y	Y	Y	Y	Y
Barbagallo et al. (2020)	Y	Y	Y	Y	Y	Y	Y
Chen et al. (2017)	Y	Y	N	U	Y	Y	Y

Y, Yes; U, Unclear; N, No.

### Pilot study: samples validation

**Figure 4:**
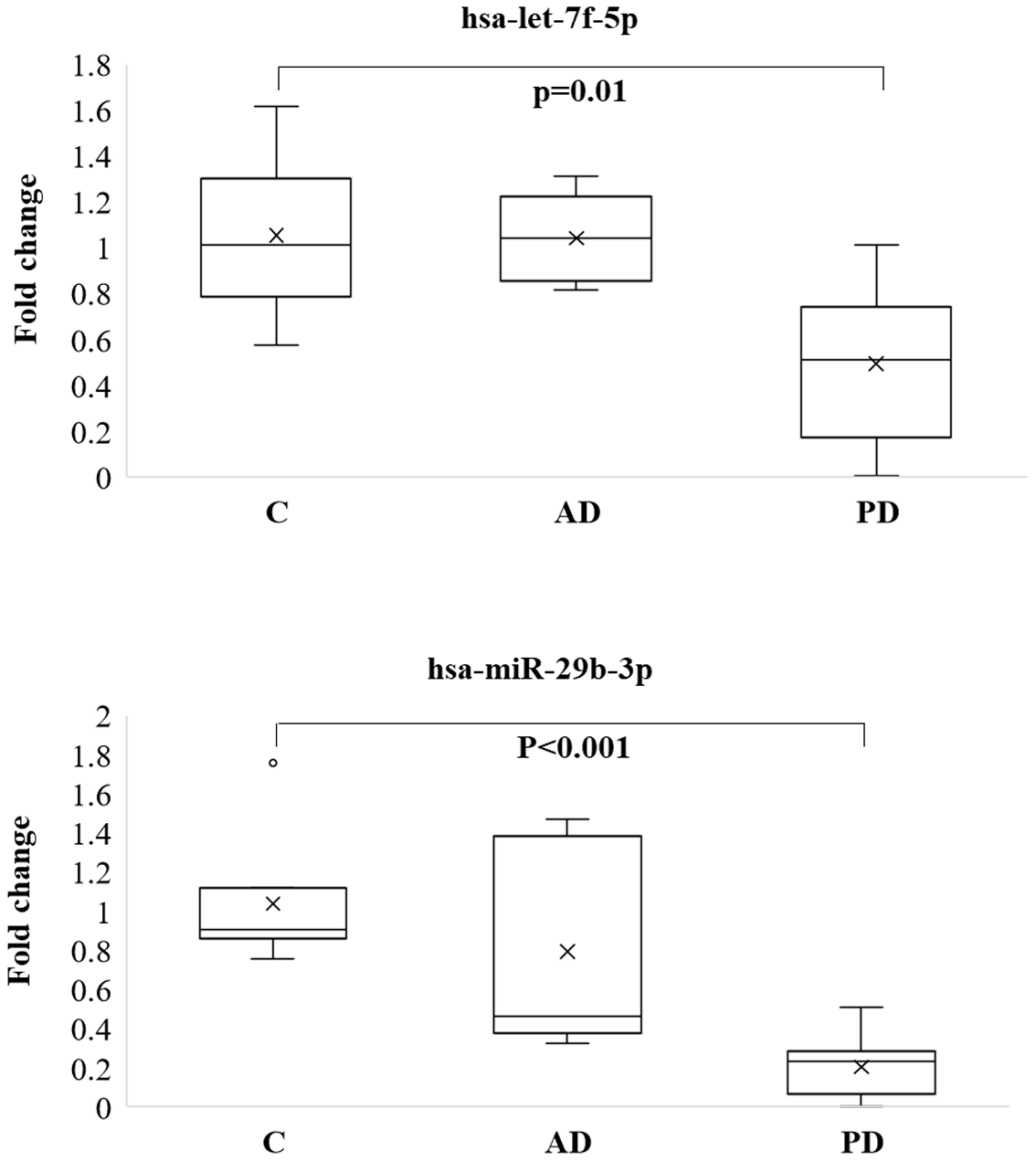
Box plots of let-7f-5p and miR-29b-3p analyzed by qPCR in blood of 5 AD and 10 PD patients and compared to 7 controls.

Let-7f-5p and miR-29b-3p were selected for the pilot study, among the common miRNAs in 2 pathologies and their expression was analyzed in 5 whole blood samples of AD patients and 10 whole blood samples of PD patients, both groups compared to control (C) whole blood samples (N = 7). Both miRs did not show any significant expression in AD samples compared to controls, while both were significantly downregulated in PD showing a *p* value of 0.01 and < 0.001, respectively, (Figure 4). One-way ANOVA with post-hoc Tukey’s multiple comparison test was also performed among the 3 different groups and showed adjusted value of *p*s of 0.997, 0.005, and 0.013 when let-7f was compared between CvsAD; CvsPD; and AD vs PD, respectively. Same analysis was performed for miR-29b-3p and showed adjusted value of *p*s of 0.434; 0.0002; 0.010 for the same comparisons.

## Conclusion

MiRNAs are non-coding RNAs whose function is to regulate the expression of genes via miRNA-induced silencing complex, in both physiological and pathological conditions and including neurological diseases. The ability of miRNA to regulate several physiological pathways makes it appealing in revealing new molecular mechanisms and suggesting new potential treatments. The aim of this work was to identify common miRNAs in 2 of the most important neurodegenerative diseases, potentially unveiling pathways that may be affected by both disorders and determining the link that irreversible lead to neurodegeneration.

In this study we have identified 15 common miRNAs. Five of them showed dysregulation in opposite direction in the 2 pathologies, suggesting a potential different response in the same altered pathway. Interestingly, our data showed the fatty acid (FA) biosynthesis, metabolism and degradation among the most highly significant and dysregulated pathways in both conditions. This is not surprising as brain is the second most lipid-rich organ in the body, contributing to many fundamental cellular processes, such as membrane synthesis, energy storage, signaling, and complex protein modifications (Sastry, 1985), therefore, FA homeostasis is an essential determinant of neural development, neurotransmission, and receptor activation. In AD research, cholesterol was demonstrated to contribute to the development of amyloid plaques by facilitating the formation of β-sheets (Zhou and Xu, 2012), as well as decreased concentrations of docosahexaenoic acid (DHA) were demonstrated to contribute to increased production of Amyloid β (Grimm et al., 2011).

In PD also, a disruption of FA homeostasis was reported, suggesting a role of polyunsaturated fatty acids in the formation of Lewy bodies, through the interactions of the polyunsaturated fats and the N-terminal of α-synuclein forming oligomers (Karube et al., 2008). Furthermore, a previous study supported that polyunsaturated and saturated fatty acids stabilize soluble α-synuclein oligomers (Sharon et al., 2003).

Another importantly compromised pathway that was identified by our bioinformatic analysis, is the extracellular matrix (ECM) receptor interaction pathway. ECM, which is estimated to constitute 20% of the brain, is involved in essential roles such as cell migration, proliferation and differentiation. This pathway is known to be affected in both AD and PD (Lam et al., 2019; Crapser et al., 2020) and glycosaminoglycans (GAGs), a component of the ECM, are seen in Lewy bodies and might play a role in accumulating α-synuclein by impeding its degradation and thereby facilitating Lewy bodies’ formation (Raghunathan et al., 2020).

Another pathway related to the ECM is the focal adhesion pathway which was also identified as a commonly dysregulated pathway. Focal adhesions play an essential role in cell migration, allowing cells to respond to stimuli. Under normal conditions, focal adhesion kinases (FAK) signal regulate the formation of these adhesions, and the focal adhesion molecules interact with the ECM through integrins whose activation may assist amyloid β plaque formation (Wright et al., 2007). Another study (Wang Q. et al., 2008) showed that αv integrins could also cause inhibition of long-term potentiation, disrupting synaptic plasticity and resulting in neurodegenerative effects.

Within PD, there is less evidence for the involvement of focal adhesions in pathogenesis. However, focal adhesions are critical to the neuronal interactions between other neurons and their environment. The cell adhesion pathway is hypothesized to be disrupted in PD, which affects the interaction with synaptic vesicles (Chapman, 2014). The rapid firing nature of dopaminergic neurons in the substantia nigra creates a greater demand for neurotransmitter release from the vesicles; therefore, this hypothesized dysfunction profoundly impacts neuronal communication. This process is believed to be affected by actin as actin interacts with vesicles and affects their fusion to the synaptic membrane. In addition, microtubules are thought to be involved in the trafficking of vesicles between neurons, affecting neuronal communication (Chapman, 2014). There is evidence that synaptic dysfunction occurs early in PD with decreased dopamine synthesis, storage and release; therefore, these hypothesized mechanisms could be involved in the pathogenesis of PD (Nikolaus et al., 2009). Nonetheless, further research is needed into the potential impact of the focal adhesion pathway on the pathology of PD.

Among the common list of differentially regulated miRNAs, in our pilot study we chose to validate, in a small cohort of patients, two miRNAs, let-7f-5p and miR-29b-3p and explore their potential role as non-invasive biomarkers. Let-7f-5p showed a different trend in the 2 diseases, being upregulated in AD and downregulated in PD (Hara et al., 2017; Chen et al., 2018), its target genes are involved in the cell cycle, apoptosis and cell adhesion (Ghanbari et al., 2015). However, the mechanisms through which let-7f-5p plays a role in pathogenesis are yet to be understood. An overexpression of let-7f-5p was seen in cells undergoing oxidative damage *in vitro* (Li K. et al., 2020). In this study, the authors using let-7f mimic have found the viability of the cells undergoing oxidative stress to improve while also decreasing apoptosis. It was discovered that AKT-2 can be repressed by let-7f, which is involved in the PI3K-Akt pathway, affecting cell proliferation and apoptosis (Li K. et al., 2020). Through this mechanism, it is speculated that the hsa-let-7f mimic can improve cell viability and reduce apoptosis.

Let-7f-5p also has a role in inflammation, targeting NLRP3 and pro-IL-1β, and repressing their expression (Tan et al., 2019). MiR-29b-3p, instead, is highly expressed in the brain and spinal cord (Smirnova et al., 2005; Hébert et al., 2008). It is involved in different mechanisms (extracellular matrix, insulin signaling, angiogenesis) (Cushing et al., 2011; Yang et al., 2014; Zhang et al., 2014) and regulates distinct cell population or pathologies (Park et al., 2009; Kwon et al., 2019) can also play crucial role during neuronal maturation, or can target BH3 protein levels in favor of neuronal degeneration (Liu et al., 2015; Huang et al., 2018). Its downregulation has been previously described in peripheral blood mononuclear cells (Villa et al., 2013), plasma (Lugli et al., 2015) and brain of subjects(Hébert et al., 2008) with AD whereby up-regulation has been seen in the cerebrospinal fluid of AD patients (Kiko et al., 2014). In addition, positive correlations were described between miR-29b concentration in serum and cortical thickness and cortical glucose metabolism (Maldonado-Lasuncion et al., 2019), which both decrease in AD. Finally, there are also studies showing the use of miR-29b as potential therapeutic agent in AD (Pereira et al., 2017).

Our data, despite the small sample size, support the existing literature, showing the downregulation of both miRNAs, let-7f and miR-29b in blood of PD patients and therefore confirming their use as potential biomarkers. On the contrary, both microRNA did not show any significant results in AD patients, although several limitations must be noted.

First of all, samples size is minimal, the number of patients that composes the validation cohorts is very low, especially for AD cohort. Moreover, AD patients were all diagnosed with an early stage of the disease and previous work showed a less strong signature of miRNA profile in the initial phase (Watson et al., 2022). Hence the large standard deviation and the consequent low statistical power for the validation studies. Therefore, a larger cohort is necessary to validate these findings.

Furthermore, there is an evident lack of age matching that may confound the interpretation of miRNA data. Therefore an age-matched control group is required for this study. The samples were extracted from whole blood, and most of the studies in the SRs reported the expression in serum/plasma. Therefore, the miRNA expression is influenced by intracellular miRNA-content. Finally, only 2 miRNAs were selected and tested in this pilot study. These two were selected on the basis of our previous results showing potential long-term implications in neurodegenerative process after a brain injury (Pietro et al., 2021).

In conclusion, this study did identify possible ways in which AD and PD share similar pathways leading to pathology, providing potential targets for future research into the disease and its treatments. A crosstalk interaction study between miRNAs and their targets related to the identified pathways is now necessary in order to propose potential area of intervention. Finally, this work was also able to show the use of miR-29b-3p as a non-invasive biomarker for PD.

## Materials and methods

### Study design

Two parallel and independent systematic reviews were undertaken using the Preferred Reporting Items for Systematic Reviews and Meta-Analyses (PRISMA) guidelines (Liberati et al., 2009). The research question is as follows: “To identify the differentially expressed miRNAs in blood/serum/plasma of Alzheimer’s and Parkinson’s diseases.” From the research question focused keywords were used as search terms in order to gather relevant records from three databases. Records eligible for inclusion were deciphered through a strict inclusion and exclusion criteria. The inclusion and exclusion criteria’s were used to complete an abstract and full-text screening. This was carried out manually by a primary reviewer and then included articles were checked by a second reviewer (B.W., V.DP.). Data extracted and miRNAs identified through the literature search were entered into DIANA database, for *in silico* bioinformatic analysis of predicted gene targets and Kyoto Encyclopedia of Genes and Genomes (KEGG) pathway identification. List of predicted targets were further evaluated by ShinyGo 0.77 for Gene Ontology (GO) enrichment analyses.

Two common miRNAs were selected from this review and used for a pilot study. Let-7f-5p and miR-29b-3p were analyzed in whole blood of 5 AD and 10 PD patients to confirm their validity as potential blood biomarkers.

### Search strategy

The Population, Intervention, Comparison (s), and Outcome (s) (PICOs) framework was used to determine a focused research question for the systematic review. This Cochrane Collaboration-recommended system enables a quantitative analysis of the results by the comprehensive gathering of the evidence within the defined parameters.

#### Population

Patients over 60 diagnosed with AD and PD of all races and genders.

Cut-off of 60 years old was chosen, since a stronger miRNA signature is identified in late AD stage (Watson et al., 2022). While people with Parkinson’s are generally diagnosed at an average age of 60 (Samii et al., 2004).

#### Intervention

Patients have received an AD or PD diagnosis against the clinical criteria.

#### Comparison

Healthy age-matched individuals who do not meet the clinical requirements for AD, PD or cognitive impairment diagnosis.

#### Outcome

The dysregulation of miRNA in serum/plasma/blood of AD and PD compared to the healthy controls to identify potential common pathways.

### Search terms and database

In order to comply with PRISMA guidelines, a selection of keywords were created. To ensure all relevant titles were covered, all possible spellings and abbreviations were used as keywords. These were searched using three different databases, PubMed, EMBASE and Web of Science. Separate searches were done for papers concerning PD and AD using separate keywords. The keywords used are as follows: “Alzheimer’s disease” AND “microRNA” OR “miRNA” OR “miR” AND “dysregulation” OR “Upregulation” OR “downregulation” AND “human” AND “blood” OR “plasma” OR “serum.” For the PD search, the keywords used are as follows: “Parkinson’s disease” AND “microRNA” OR “miRNA” OR “miR” AND “dysregulation” OR “upregulation” OR “downregulation” AND “human” AND “blood” OR “plasma” OR “serum.” The records retrieved were collated in Endnote 20 (Clarivate, Philadelphia, PA, USA) where duplicates were screened and any identified were removed. Using the defined inclusion and exclusion criteria, the remaining abstracts were then manually evaluated. Two independent reviewers evaluated the eligible records as outlined in Table 6. Papers analyzed were filtered between 2011 and Feb 2022 to guarantee that all searches were up to date with research.

**Table 6:** Inclusion and exclusion criteria utilized to direct the miRNA in AD and PD systematic literature search.

Inclusion	Exclusion
Confirmed clinical diagnosis of Alzheimer’s or Parkinson’s diseases	Probable or possible AD/PD and other neurodegenerative diseases and cognitive impairment
Blood/plasma/serum samples	Post-mortem brain sample, urine sample, saliva samples
qRT-PCR, RNAseq, microarray analysis	*in-situ* hybridization, transfection and functional study
Qualitative and quantitative analysis	Study focusing on post-translational modifications, mutations, allelic variants, study including treatment or intervention
Human	Animals, cell lines
Male and female participants	None
Age-matched controls compared to AD	Single cohort studies, case studies, non-age-matched controls
Age ≥ 60	Age < 60
All patient ethnicities	No ethnicities were excluded
Primary research	Reviews, meta-analyses, bioinformatics studies using previously collected data, conference abstracts, clinical trials
Sample size *n* ≥ 3	Sample size *n* < 3
Published in peer-reviewed journals	Non-peer-reviewed
English language	Not written in English

### Data collection

After screening, the papers that fit the inclusion criteria were saved into a separate excel file. The excel datafile included the title, the authors, the year published, and the URL. Two separate files were made for the papers describing AD and those describing PD. Then the text was screened further to identify the population of those with AD/PD and the controls, the gender, age, and AD or PD severity. Further, the miRNAs were identified, whether they were up or downregulated, and lastly, the technique used was identified. This step ensured that the relevant information from each study was included. Data were extracted from the final included studies and imported into Microsoft Excel (Microsoft Corporation, Redmond, WA, USA).

### Quality appraisal

A critical appraisal was conducted using the JBI checklist tool for systematic reviews to ensure the studies quality and determine if there was any bias (Brackett and Batten, 2022). The JBI checklist included seven questions that were answered with either yes, no, unclear or not applicable. Q3 “Exposure measures in a valid and reliable manner” has been removed since it is not applicable to this study. The articles were then given a quality score calculated by (number of yes responses/7) *100. For this study, the quality included was greater than 60%.

### Pathway analysis

Pathway analysis was performed using DIANA tools miRpaths v.3 to search the associated pathways extensively^1^ (Paraskevopoulou et al., 2013). By using this system, the significantly associated miRNA-regulated pathways were identified. The miRNAs identified through the systematic review were added to the system independently. The bioinformatic analysis was done separately for the miRNAs reported in AD and PD papers. Then the identified common miRNAs were also inputted into the system to analyze the common pathways affected. Based on the DIANA-micro-T-CDS algorithm, miRNA-mRNA interactions were predicted in silicon (Vlachos et al., 2015).

List of predicted targets was further evaluated using ShinyGO 0.77 software for enriched pathway and GO analyses^2^ (Luo and Brouwer, 2013; Ge et al., 2020; Kanehisa et al., 2021).

### Pilot study: sample validation

The study was carried out in accordance with the recommendations of the University of Birmingham Research Ethics Committee (Ethics Ref 18-315). All participants gave written informed consent in accordance with the Declaration of Helsinki. Participants were consented for blood samples from clinical staff from the Human Biomaterials Resource Center, University of Birmingham, at routine clinical appointments undertaken at the Queen Elizabeth Hospital Birmingham. Blood samples were collected in EDTA and frozen whole at-80c until analysis. Male and female participants with a confirmed diagnosis of AD or PD, were enrolled in this study. *Diagnosis was made on individual’s history, symptoms, physical exam and evaluation of* Mini-Mental State Examination (MMSE). Demographic data is detailed in Table 7.

**Table 7:** Demographic data of patients recruited in this study.

Group	*N*	Age (average ± SD)	Gender M (Age average ± SD):F (average ± SD)	Ethnicity
Controls (C)	7	65 ± 6	3 (65.5 ± 2.1):4 (62 ± 0.8)	5 White-British; 1 not specified; 1 Black Caribbean
AD	5	79 ± 11	3 (74.2 ± 8.9): 2 (87 ± 2.8)	2 White-British; 3 not specified
PD	10	68 ± 14	5 (64.8 ± 14.3): 5 (70.4 ± 14.6)	6 White-British; 3 not specified; 1 Black Caribbean

### RNA isolation

Total RNA was isolated from 200 μL of whole blood by using Qiagen miRNeasy Mini Kit (Qiagen, GmbH, Hilden, Germany), according to Qiagen Protocol. RNA was quantified using a NanoDrop^®^ ND-1000 spectrophotometer (Thermo Scientific, Wilmington, DE, United States). An Agilent 2,100 Bioanalyzer (Santa Clara, CA, United States) was used to detect the size distribution of total RNA, as well as determine the quality of the RNA.

### Single TaqMan assays

Two differentially expressed miRNA were chosen among the common miRNAs of the systematic reviews. Samples were analyzed by single TaqMan assays (Applied Biosystems, Life Technologies™). Samples were retrotranscribed (Applied Biosystems, Life Technologies™) and RT-qPCR analysis was performed in a Bio-Rad iQ5 Real-time PCR Detection System (Bio-Rad, CA, United States). Expression fold changes were calculated by the 2^-ΔΔCT^ method by using miR-16 as reference gene (Shahid et al., 2019).

### Statistical analysis

A non-parametric test (Mann–Whitney *U* test) was used to compare the level of miRNAs in the independent groups (C vs. AD; C vs. PD). A *value of p* <0.05 was accepted as significant. One-way Anova with post-hoc *Tukey* for multiple comparison analysis for the 3 groups was also performed. All statistical analyses were carried on SPSS v.22 (IBM).

## Data availability statement

The original contributions presented in the study are included in the article/Supplementary material, further inquiries can be directed to the corresponding author.

## Ethics statement

The studies involving humans were approved by the study was carried out in accordance with the recommendations of the University of Birmingham Research Ethics Committee (Ethics Ref 18-315). The studies were conducted in accordance with the local legislation and institutional requirements. The participants provided their written informed consent to participate in this study.

## Author contributions

AW, LH, and VD: conceptualization and resources. BA-D, LH, and VD: methodology, formal analysis, data curation, and writing – original draft preparation. VD: validation. BW and AW: writing – review and editing. LH: project administration. LH and VD: funding acquisition. All authors contributed to the article and approved the submitted version.

## Funding

This project was funded by The Midland Neuroscience Teaching and Research Fund (Charity number 313446), Queen Elizabeth Hospital Birmingham.

## Conflict of interest

The authors declare that the research was conducted in the absence of any commercial or financial relationships that could be construed as a potential conflict of interest.

## Publisher’s note

All claims expressed in this article are solely those of the authors and do not necessarily represent those of their affiliated organizations, or those of the publisher, the editors and the reviewers. Any product that may be evaluated in this article, or claim that may be made by its manufacturer, is not guaranteed or endorsed by the publisher.

## Footnotes

^1^http://snf-515788.vm.okeanos.grnet.gr/is last accessed 13 March 2022.

^2^http://bioinformatics.sdstate.edu/go/ last accessed 19 May 2023.
