# Supplementary material for: Common microRNA regulated pathways in Alzheimer’s and Parkinson’s disease
Source: Front Neurosci. 2023 Sep 1;17:1228927. doi: 10.3389/fnins.2023.1228927 (PMC10502311; doi:10.3389/fnins.2023.1228927)
Supplement: Supplementary file 1 [file Table_1.pdf]

Supplementary Table 1: The miRNAs obtained from the search regarding AD resulted in 162 KEGG pathways listed below. FDR adjusted P-values, number of genes and number of miRNAs related to each pathway are also reported.

KEGG pathway	FDR adjusted p-value	#genes	#miRNAs
TGF-beta signaling pathway	3.10E-13	50	19
Pancreatic secretion	0.00652	4	1
Calcium signaling pathway	0.042659	4	1
Cell adhesion molecules (CAMs)	9.19E-09	23	3
Cardiac muscle contraction	0.016852	9	2
Proteoglycans in cancer	4.22E-07	114	19
Glycosphingolipid biosynthesis - lacto and neolacto series	5.10E-12	14	10
Glycosaminoglycan biosynthesis - heparan sulfate / heparin	0.000157	11	8
Glycosphingolipid biosynthesis - ganglio series	0.000253	7	5
Pathways in cancer	1.50E-06	173	10
Metabolism of xenobiotics by cytochrome P450	9.87E-15	8	6
Amphetamine addiction	0.002173	35	10
Arrhythmogenic right ventricular cardiomyopathy (ARVC)	0.000263	31	4
Amyotrophic lateral sclerosis (ALS)	0.014017	10	2
Ubiquitin mediated proteolysis	1.48E-09	79	11
Morphine addiction	1.29E-08	35	11
Glycosaminoglycan biosynthesis - keratan sulfate	0.019625	6	4
FoxO signaling pathway	6.58E-06	68	12
Gap junction	4.57E-05	39	12
cGMP-PKG signaling pathway	0.009827	66	6
Axon guidance	9.88E-05	79	16
Mucin type O-Glycan biosynthesis	1.23E-20	22	21
B cell receptor signaling pathway	0.009455	13	2
Cocaine addiction	3.38E-05	19	7
Long-term depression	1.38E-06	24	8
Transcriptional misregulation in cancer	1.02E-06	81	10
mTOR signaling pathway	0.004778	20	4
Fatty acid biosynthesis	4.22E-39	5	5
Melanoma	0.00035	35	6
Glioma	0.000118	32	14
Insulin signaling pathway	0.032326	25	2
Oocyte meiosis	0.000233	31	5
Hippo signaling pathway	2.33E-15	86	11
Fatty acid metabolism	5.57E-16	15	7
Colorectal cancer	5.88E-05	34	8
Non-small cell lung cancer	0.011928	16	4
Wnt signaling pathway	1.25E-06	79	10
AMPK signaling pathway	0.002767	54	5
Signaling pathways regulating pluripotency of stem cells	5.21E-11	99	18
Thyroid cancer	0.022087	8	3

Progesterone-mediated oocyte maturation	0.004778	22	4
Prostate cancer	0.000285	43	4
PI3K-Akt signaling pathway	3.01E-05	76	3
Focal adhesion	1.47E-06	104	6
Pancreatic cancer	0.016827	20	3
p53 signaling pathway	0.002183	30	3
ECM-receptor interaction	########	45	15
Lysine degradation	2.69E-08	23	13
Vitamin B6 metabolism	0.044105	1	2
Thyroid hormone signaling pathway	6.58E-06	73	13
ErbB signaling pathway	0.007812	18	4
Mineral absorption	0.038013	8	2
Circadian rhythm	0.002767	19	9
Neurotrophin signaling pathway	0.011057	20	5
Ras signaling pathway	0.012827	69	5
Hepatitis B	0.00042	32	7
MAPK signaling pathway	0.001438	86	7
Endocytosis	5.45E-05	67	8
Protein processing in endoplasmic reticulum	0.00033	66	8
Chronic myeloid leukemia	0.010695	30	5
Oxytocin signaling pathway	0.030945	15	1
Hedgehog signaling pathway	0.001325	30	6
Retrograde endocannabinoid signaling	0.012463	22	4
GABAergic synapse	0.0078	17	4
Adrenergic signaling in cardiomyocytes	0.000404	35	4
Inositol phosphate metabolism	0.038666	22	5
Pantothenate and CoA biosynthesis	0.006547	4	3
Salivary secretion	0.040877	12	1
cAMP signaling pathway	0.009655	63	3
Dopaminergic synapse	0.009655	27	2
Vascular smooth muscle contraction	0.032326	16	1
Glycerophospholipid metabolism	0.045228	19	2
Prolactin signaling pathway	0.009429	21	4
Estrogen signaling pathway	0.001526	26	6
Maturity onset diabetes of the young	0.000316	7	3
Adherens junction	5.02E-05	38	8
Endocrine and other factor-regulated calcium reabsorption	0.000335	17	7
Renal cell carcinoma	1.50E-06	37	5
2-Oxocarboxylic acid metabolism	0.001159	6	4
Melanogenesis	0.02857	11	1
Vasopressin-regulated water reabsorption	0.00044	9	2
Glutamatergic synapse	0.001068	23	4
Ovarian steroidogenesis	0.012463	2	2
Ubiquinone and other terpenoid-quinone biosynthesis	0.018433	1	2
Circadian entrainment	7.96E-06	16	5
Alcoholism	0.042243	3	2

Drug metabolism - cytochrome P450	4.71E-19	10	2
Chemical carcinogenesis	0.005057	3	2
D-Glutamine and D-glutamate metabolism	0.030999	1	1
Rap1 signaling pathway	0.001638	62	2
Phosphatidylinositol signaling system	0.002767	27	4
Basal cell carcinoma	0.008061	30	5
Biotin metabolism	4.57E-09	1	3
Dorso-ventral axis formation	0.000777	15	2
Regulation of actin cytoskeleton	0.02796	70	3
Sphingolipid signaling pathway	0.016704	40	4
Viral carcinogenesis	0.019492	57	6
N-Glycan biosynthesis	0.000784	19	6
Bacterial invasion of epithelial cells	0.002956	26	2
mRNA surveillance pathway	0.001663	41	4
Lysine biosynthesis	0.012313	1	1
Purine metabolism	4.68E-05	12	2
Sulfur metabolism	0.029536	3	2
Allograft rejection	4.63E-05	5	2
Acute myeloid leukemia	0.00222	20	4
Autoimmune thyroid disease	0.014521	4	1
Antigen processing and presentation	0.00868	5	2
Vitamin digestion and absorption	0.018719	4	2
Other glycan degradation	1.46E-07	3	4
Valine, leucine and isoleucine degradation	0.00892	5	2
Glycosphingolipid biosynthesis - globo series	0.036926	4	2
Biosynthesis of unsaturated fatty acids	2.68E-14	5	7
Hematopoietic cell lineage	0.013335	5	2
Cell cycle	0.036999	5	1
RNA transport	0.017077	16	3
Nicotinate and nicotinamide metabolism	0.005465	5	2
Terpenoid backbone biosynthesis	0.03091	2	1
MicroRNAs in cancer	0.008561	35	3
Glycosaminoglycan biosynthesis - chondroitin sulfate / dermatan sulfate	0.000879	5	4
Amoebiasis	2.30E-17	24	3
Long-term potentiation	0.047643	18	3
Cytokine-cytokine receptor interaction	0.036407	5	2
D-Arginine and D-ornithine metabolism	0.000448	1	2
Phototransduction	0.02727	1	1
Thyroid hormone synthesis	7.09E-13	7	6
Caffeine metabolism	0.01573	1	2
Steroid biosynthesis	4.12E-07	1	2
Bile secretion	0.005715	1	1
Endometrial cancer	0.006547	12	3
Prion diseases	1.30E-33	3	3
Pyrimidine metabolism	0.005895	1	1
Folate biosynthesis	4.57E-05	1	1

Nucleotide excision repair	0.03941	3	1
Proteasome	0.038378	1	1
One carbon pool by folate	0.039378	2	1
Phagosome	0.033255	6	1
Base excision repair	0.006318	3	1
Fatty acid degradation	4.57E-12	3	2
Ether lipid metabolism	0.045228	4	1
Amino sugar and nucleotide sugar metabolism	0.002241	8	2
Steroid hormone biosynthesis	6.15E-13	7	1
Ascorbate and aldarate metabolism	0.021336	7	1
Porphyrin and chlorophyll metabolism	0.021336	9	1
Synaptic vesicle cycle	0.00755	4	1
Shigellosis	0.031888	4	1
Valine, leucine and isoleucine biosynthesis	0.008391	1	1
Inflammatory mediator regulation of TRP channels	0.030114	4	1
Small cell lung cancer	6.36E-05	15	1
Glycosaminoglycan degradation	0.003832	3	1
Protein digestion and absorption	7.13E-11	23	1
Platelet activation	0.004987	13	1
Sulfur relay system	0.012847	1	1
Tyrosine metabolism	0.000393	3	1
Central carbon metabolism in cancer	0.000195	9	2
Metabolic pathways	0.039434	45	1
Hypertrophic cardiomyopathy (HCM)	0.046194	9	1
Apoptosis	0.027678	1	1
Tuberculosis	0.027678	1	1
Spliceosome	0.027678	1	1
NF-kappa B signaling pathway	0.004916	3	1
HIF-1 signaling pathway	0.013912	4	1
Fructose and mannose metabolism	0.030625	2	1
