# Supplementary material for: Common microRNA regulated pathways in Alzheimer’s and Parkinson’s disease
Source: Front Neurosci. 2023 Sep 1;17:1228927. doi: 10.3389/fnins.2023.1228927 (PMC10502311; doi:10.3389/fnins.2023.1228927)
Supplement: Supplementary file 2 [file Table_2.pdf]

Supplementary Table 2: The miRNAs obtained from the search regarding PD resulted in 146 KEGG pathways listed below. FDR-adjusted P-values, number of genes and number of miRNAs related to each pathway are also reported.

**KEGG pathway**	**FDR-adjusted p-value**	**#genes**	**#miRNAs**
Proteoglycans in cancer	0.000120492	122	23
ECM-receptor interaction	6.21E-124	47	23
Melanoma	0.000413321	36	9
Glioma	1.82E-06	41	20
Small cell lung cancer	2.99E-05	16	7
Lysine degradation	9.04E-06	23	15
Amoebiasis	1.05E-17	24	9
Thyroid hormone signaling pathway	0.000541041	46	8
Choline metabolism in cancer	0.034350411	23	3
Non-small cell lung cancer	0.011010861	23	8
Glycosaminoglycan biosynthesis - heparan sulfate / heparin	1.61E-07	8	16
Estrogen signaling pathway	5.63E-08	32	8
Protein digestion and absorption	4.28E-11	23	7
Prostate cancer	0.000343962	46	9
PI3K-Akt signaling pathway	1.69E-05	158	16
Focal adhesion	8.68E-07	100	12
Platelet activation	0.003679457	14	7
Pancreatic cancer	0.00598842	14	7
Oxytocin signaling pathway	0.018001541	24	2
Arachidonic acid metabolism	0.000434913	3	1
Glycosphingolipid biosynthesis - lacto and neolacto series	9.08E-27	12	13
Transcriptional misregulation in cancer	5.37E-06	68	8
Prion diseases	3.20E-44	1	2
Sulfur metabolism	0.000485635	2	3
Hedgehog signaling pathway	0.027010263	7	3
Central carbon metabolism in cancer	0.048657872	5	2
Synaptic vesicle cycle	0.048657872	4	2
Vitamin digestion and absorption	0.012499	4	3
Glycosaminoglycan degradation	0.003832074	3	3
Metabolism of xenobiotics by cytochrome P450	0.004172543	5	7
Maturity onset diabetes of the young	0.003088036	1	2
Type II diabetes mellitus	0.003088036	1	2
Systemic lupus erythematosus	8.89E-05	17	1
Steroid hormone biosynthesis	6.81E-05	4	2
Hippo signaling pathway	3.05E-05	72	12
Alcoholism	6.81E-05	18	1
Other glycan degradation	8.89E-05	2	1
Thyroid hormone synthesis	1.55E-09	16	4

Amphetamine addiction	0.000128499	34	10
Arrhythmogenic right ventricular cardiomyopathy (ARVC)	1.99E-05	29	4
ErbB signaling pathway	0.001341209	46	11
TGF-beta signaling pathway	4.90E-05	48	16
Ubiquitin mediated proteolysis	0.001207932	54	2
Phosphatidylinositol signaling system	0.001928984	36	7
Biotin metabolism	7.29E-16	1	5
Gap junction	0.003546798	39	7
Viral carcinogenesis	0.034835508	48	3
Nicotine addiction	0.000418876	19	8
Ras signaling pathway	0.009659912	75	4
Renal cell carcinoma	0.040045327	23	2
Signaling pathways regulating pluripotency of stem cells	1.65E-07	82	17
Biosynthesis of unsaturated fatty acids	0.003346174	8	2
Endometrial cancer	0.002830081	23	3
Mucin type O-Glycan biosynthesis	2.52E-06	15	17
HIF-1 signaling pathway	0.040045327	27	1
Pathways in cancer	0.001028393	142	8
Glycosphingolipid biosynthesis - globo series	0.018166558	4	2
Sphingolipid signaling pathway	0.017538336	22	2
MicroRNAs in cancer	0.000534487	20	1
SNARE interactions in vesicular transport	0.023035281	5	1
Inflammatory mediator regulation of TRP channels	0.020222862	23	3
Long-term potentiation	0.033245813	23	5
Morphine addiction	0.000865618	33	9
Circadian entrainment	0.00013857	32	6
Wnt signaling pathway	0.000585624	52	9
Vasopressin-regulated water reabsorption	7.11E-05	6	3
Intestinal immune network for IgA production	0.044903628	2	1
Base excision repair	0.024101274	1	1
Terpenoid backbone biosynthesis	0.030910486	4	3
Adrenergic signaling in cardiomyocytes	0.000404045	31	5
FoxO signaling pathway	3.38E-05	45	7
AMPK signaling pathway	0.007782869	22	6
Axon guidance	0.000434329	66	11
Dopaminergic synapse	0.004055926	35	3
Cardiac muscle contraction	0.016656479	7	1
Cell adhesion molecules (CAMs)	0.035253605	14	6
Adherens junction	0.000178174	17	3
Bacterial invasion of epithelial cells	0.00051264	22	3
Glutamatergic synapse	1.99E-05	41	8
Glycosaminoglycan biosynthesis - chondroitin sulfate / dermatan sulfate	0.000879394	3	2
p53 signaling pathway	0.002086554	21	6
Adipocytokine signaling pathway	0.015522616	8	1

Serotonergic synapse	0.025277348	17	3
GABAergic synapse	0.012312575	17	6
Cocaine addiction	0.003507263	7	4
Long-term depression	0.000866823	24	10
mTOR signaling pathway	0.001278863	23	7
Ovarian steroidogenesis	0.043327718	6	2
Rap1 signaling pathway	0.003546798	22	2
Prolactin signaling pathway	0.004055926	29	11
Gastric acid secretion	0.034484564	11	2
GnRH signaling pathway	0.020222862	12	3
Progesterone-mediated oocyte maturation	0.004919505	25	7
Salivary secretion	0.023032332	12	3
cAMP signaling pathway	0.005458099	42	3
Glycosphingolipid biosynthesis - ganglio series	7.79E-07	6	4
Hepatitis B	0.00957831	4	1
Purine metabolism	0.018582917	8	3
Fatty acid biosynthesis	4.61E-39	4	8
One carbon pool by folate	0.010740372	1	1
Folate biosynthesis	0.0076459	1	1
cGMP-PKG signaling pathway	0.010647797	50	4
Endocrine and other factor-regulated calcium reabsorption	0.00858921	9	1
Fatty acid degradation	4.57E-12	4	5
Circadian rhythm	0.038149648	4	4
Fatty acid metabolism	6.60E-10	10	10
Insulin signaling pathway	0.046720441	17	4
Oocyte meiosis	0.000448352	18	6
Colorectal cancer	0.013999244	10	5
Thyroid cancer	0.00598842	6	5
Pyrimidine metabolism	0.016151103	1	2
Sphingolipid metabolism	0.007628389	3	4
Retrograde endocannabinoid signaling	0.014184325	6	1
Amino sugar and nucleotide sugar metabolism	0.002667418	8	2
Valine, leucine and isoleucine degradation	0.031986507	1	1
Chronic myeloid leukemia	0.039773611	16	2
Fatty acid elongation	0.000344037	1	1
Glycosaminoglycan biosynthesis - keratan sulfate	0.002967758	4	3
Other types of O-glycan biosynthesis	0.019660576	2	2
Huntington's disease	1.26E-07	13	2
Amyotrophic lateral sclerosis (ALS)	0.014017295	10	1
B cell receptor signaling pathway	0.009454959	13	1
RNA degradation	0.008086841	7	1
Pantothenate and CoA biosynthesis	0.045007468	2	1
Tight junction	0.045007468	8	1
Antigen processing and presentation	0.04241092	5	1
Inositol phosphate metabolism	0.03950628	3	1
TNF signaling pathway	0.015141783	3	1

Proteasome	0.015141783	2	1
Vascular smooth muscle contraction	0.000983241	6	2
N-Glycan biosynthesis	9.31E-05	8	3
Alanine, aspartate and glutamate metabolism	0.018420548	2	1
Protein processing in endoplasmic reticulum	0.046095913	5	1
Cholinergic synapse	0.018001541	17	1
Neurotrophin signaling pathway	0.009659912	18	1
Regulation of actin cytoskeleton	0.01817098	35	2
Cytokine-cytokine receptor interaction	0.036406957	6	2
Mineral absorption	0.014299024	10	1
Glycerophospholipid metabolism	0.014299024	12	1
Valine, leucine and isoleucine biosynthesis	0.03080725	1	1
Hematopoietic cell lineage	0.013334648	5	1
Endocytosis	0.014362431	14	1
Pancreatic secretion	0.000983241	1	1
Insulin secretion	0.023032332	1	1
Pathogenic Escherichia coli infection	0.027010263	1	1
Basal cell carcinoma	0.027010263	1	1
