# Supplementary material for: Common microRNA regulated pathways in Alzheimer’s and Parkinson’s disease
Source: Front Neurosci. 2023 Sep 1;17:1228927. doi: 10.3389/fnins.2023.1228927 (PMC10502311; doi:10.3389/fnins.2023.1228927)
Supplement: Supplementary file 3 [file Table_3.pdf]

Supplementary Table 3: GO Biological Process

FDR	nGenes	GO terms or pathways	Description
0.0000	31	GO:0030198	Extracellular matrix organization
0.0000	27	GO:0007167	Enzyme linked receptor protein signaling pathway
0.0000	37	GO:0009888	Tissue development
0.0000	48	GO:0048513	Animal organ development
0.0000	40	GO:0009653	Anatomical structure morphogenesis
0.0000	55	GO:0048731	System development
0.0000	27	GO:0009887	Animal organ morphogenesis
0.0000	21	GO:0007169	Transmembrane receptor protein tyrosine kinase signaling pathway
0.0000	39	GO:0007166	Cell surface receptor signaling pathway
0.0000	56	GO:0007275	Multicellular organism development
0.0000	7	GO:0038063	Collagen-activated tyrosine kinase receptor signaling pathway
0.0000	57	GO:0016043	Cellular component organization
0.0000	16	GO:0009100	Glycoprotein metabolic process
0.0000	24	GO:0009790	Embryo development
0.0000	57	GO:0032502	Developmental process
0.0000	40	GO:0006464	Cellular protein modification process

FDR	nGenes	GO terms or pathways	Description
0.0000	8	GO:0030199	Collagen fibril organization
0.0000	13	GO:0006486	Protein glycosylation
0.0000	14	GO:0009101	Glycoprotein biosynthetic process
0.0000	21	GO:0072359	Circulatory system development
0.0000	16	GO:0001501	Skeletal system development
0.0000	9	GO:0006493	Protein O-linked glycosylation
0.0000	59	GO:0032501	Multicellular organismal process
0.0000	20	GO:2000145	Regulation of cell motility
0.0000	17	GO:0009792	Embryo development ending in birth or egg hatching
0.0000	15	GO:0071363	Cellular response to growth factor stimulus
0.0000	15	GO:0001568	Blood vessel development
0.0000	44	GO:0019538	Protein metabolic process
0.0000	19	GO:0030334	Regulation of cell migration
0.0000	16	GO:1901137	Carbohydrate derivative biosynthetic process
