# Supplementary material for: Common microRNA regulated pathways in Alzheimer’s and Parkinson’s disease
Source: Front Neurosci. 2023 Sep 1;17:1228927. doi: 10.3389/fnins.2023.1228927 (PMC10502311; doi:10.3389/fnins.2023.1228927)
Supplement: Supplementary file 4 [file Table_4.pdf]

Supplementary Table 4: GO Cellular components

FDR	nGenes	GO terms or pathways	Description
0.0000	22	GO:0005581	Collagen trimer
0.0000	16	GO:0098644	Complex of collagen trimers
0.0000	26	GO:0005788	Endoplasmic reticulum lumen
0.0000	26	GO:0062023	Collagen-containing extracellular matrix
0.0000	28	GO:0031012	Extracellular matrix
0.0000	9	GO:0005583	Fibrillar collagen trimer
0.0000	56	GO:0012505	Endomembrane system
0.0000	32	GO:0005783	Endoplasmic reticulum
0.0000	12	GO:0005604	Basement membrane
0.0000	7	GO:0098651	Basement membrane collagen trimer
0.0000	6	GO:0005587	Collagen type IV trimer
0.0000	19	GO:0000139	Golgi membrane
0.0000	44	GO:0005576	Extracellular region
0.0000	37	GO:0005615	Extracellular space
0.0000	74	GO:0043231	Intracellular membrane-bounded organelle
0.0000	51	GO:0070013	Intracellular organelle lumen
0.0000	79	GO:0043227	Membrane-bounded organelle

FDR	nGenes	GO terms or pathways	Description
0.0000	18	GO:0099081	Supramolecular polymer
0.0001	74	GO:0005737	Cytoplasm
0.0001	78	GO:0043229	Intracellular organelle
0.0001	8	GO:0098802	Plasma membrane signaling receptor complex
0.0001	3	GO:0005588	Collagen type V trimer
0.0001	26	GO:0098588	Bounding membrane of organelle
0.0001	22	GO:0005794	Golgi apparatus
0.0002	3	GO:0035867	alphav-beta3 integrin-IGF-1-IGF1R complex
0.0002	19	GO:0099080	Supramolecular complex
0.0004	3	GO:0048179	Activin receptor complex
0.0005	12	GO:0098797	Plasma membrane protein complex
0.0009	4	GO:0008305	Integrin complex
0.0009	82	GO:0005622	Intracellular
