# Supplementary material for: Common microRNA regulated pathways in Alzheimer’s and Parkinson’s disease
Source: Front Neurosci. 2023 Sep 1;17:1228927. doi: 10.3389/fnins.2023.1228927 (PMC10502311; doi:10.3389/fnins.2023.1228927)
Supplement: Supplementary file 5 [file Table_5.pdf]

Supplementary Table 5: GO Molecular Function

FDR	nGenes	GO terms or pathways	Description
0.0000	23	GO:0005201	Extracellular matrix structural constituent
0.0000	17	GO:0030020	Extracellular matrix structural constituent conferring tensile strength
0.0000	25	GO:0005198	Structural molecule activity
0.0000	8	GO:0048407	Platelet-derived growth factor binding
0.0000	11	GO:0019838	Growth factor binding
0.0000	12	GO:0016758	Transferase activity, transferring hexosyl groups
0.0000	6	GO:0005161	Platelet-derived growth factor receptor binding
0.0000	13	GO:0016757	Transferase activity, transferring glycosyl groups
0.0000	6	GO:0004653	Polypeptide N-acetylgalactosaminyltransferase activity
0.0000	6	GO:0018024	Histone-lysine N-methyltransferase activity
0.0000	9	GO:0008194	UDP-glycosyltransferase activity
0.0001	8	GO:0002020	Protease binding
0.0002	27	GO:0016740	Transferase activity
0.0004	22	GO:0005102	Signaling receptor binding
0.0004	26	GO:0140096	Catalytic activity, acting on a protein
0.0004	19	GO:0044877	Protein-containing complex binding

FDR	nGenes	GO terms or pathways	Description
0.0006	7	GO:0070851	Growth factor receptor binding
0.0007	3	GO:0042799	Histone methyltransferase activity (H4-K20 specific)
0.0043	5	GO:0046332	SMAD binding
0.0088	6	GO:0005178	Integrin binding
0.0134	3	GO:0004675	Transmembrane receptor protein serine/threonine kinase activity
0.0134	6	GO:0008083	Growth factor activity
0.0178	4	GO:0050840	Extracellular matrix binding
0.0262	3	GO:0005158	Insulin receptor binding
0.0270	34	GO:0046872	Metal ion binding
0.0293	4	GO:0030145	Manganese ion binding
0.0317	4	GO:0005518	Collagen binding
0.0375	2	GO:0016361	Activin receptor activity, type I
0.0375	2	GO:0034711	Inhibin binding
0.0381	3	GO:0043548	Phosphatidylinositol 3-kinase binding
